# Synergistic Regulation of Solvation Shell and Anode Interface by Bifunctional Additives for Stable Aqueous Zinc-Ion Batteries

**DOI:** 10.3390/nano15191482

**Published:** 2025-09-28

**Authors:** Luo Zhang, Die Chen, Chenxia Zhao, Haibo Tian, Gaoda Li, Xiaohong He, Gengpei Xia, Yafan Luo, Dingyu Yang

**Affiliations:** 1 Sichuan Meteorological Optoelectronic Sensor Technology and Application Engineering Research Center, Chengdu University of Information Technology, Chengdu 610225, China; 2 Information Materials and Device Applications Key Laboratory of Sichuan Provincial Universities, Chengdu University of Information Technology, Chengdu 610225, China; 3 Optoelectronic Sensor Devices and Systems Key Laboratory of Sichuan Provincial Universities, College of Optoelectronic Engineering (Chengdu IC Valley Industrial College), Chengdu University of Information Technology, Chengdu 610225, China; 4 Chengdu Product Quality Inspection and Research Institute Co., Ltd., Chengdu 610100, China; 5 POWERCHINA Chengdu Engineering Corporation Limited, Chengdu 611130, China

**Keywords:** electrolyte additives, solvation shell regulation, solid electrolyte interphase, zinc dendrites

## Abstract

Aqueous zinc-ion batteries (AZIBs) have attracted significant attention for large-scale energy storage owing to their high safety, low cost, and environmental friendliness. However, issues such as dendrite growth, hydrogen evolution, and corrosion at the zinc anode severely limit their cycling stability. In this study, a “synergistic solvation shell–interfacial adsorption regulation” strategy is proposed, employing potassium gluconate (KG) and dimethyl sulfoxide (DMSO) as composite additives to achieve highly reversible zinc anodes. DMSO integrates into the Zn^2+^ solvation shell, weakening Zn^2+^-H_2_O interactions and suppressing the activity of free water, while gluconate anions preferentially adsorb onto the zinc anode surface, inducing the formation of a robust solid electrolyte interphase (SEI) enriched in Zn(OH)_2_ and ZnCO_3_. Nuclear magnetic resonance(NMR), Raman, and Fourier transform infrared spectroscopy(FTIR) analyses confirm the reconstruction of the solvation structure and reduction in water activity, and X-ray photoelectron spectroscopy(XPS) verifies the formation of the SEI layer. Benefiting from this strategy, Zn||Zn symmetric cells exhibit stable cycling for over 1800 h at 1 mA cm^−2^ and 1 mAh cm^−2^, and Zn||Cu cells achieve an average coulombic efficiency of 96.39%, along with pronounced suppression of the hydrogen evolution reaction. This work provides a new paradigm for the design of low-cost and high-performance electrolyte additives.

## 1. Introduction

The intermittent and variable nature of renewable energy sources necessitates advanced electrochemical energy storage technologies to ensure grid stability and energy reliability [1,2]. While lithium-ion batteries dominate the current market (>85% share), their deployment in large-scale applications is hindered by safety concerns (e.g., thermal runaway), capacity degradation during cycling, and high levelized storage costs [3,4,5]. These limitations have driven intense research into alternative battery chemistries, among which aqueous zinc-ion batteries (AZIBs) have emerged as a promising candidate. AZIBs combine the high theoretical specific capacity (820 mAh g^−1^) and favorable redox potential (−0.76 V vs. SHE) of zinc metal with intrinsic safety, environmental compatibility, and abundant zinc resources—whose crustal abundance is approximately 1.5 times that of iron [6]. Despite these advantages, widespread commercialization remains impeded by three key challenges: uncontrolled zinc dendrite growth, parasitic hydrogen evolution reactions (HER), and cathode material instability during cycling [7,8].

To address these issues, researchers have proposed multiple optimization strategies for zinc anodes, primarily including zinc anode interface modification, construction of artificial protective layers, and electrolyte regulation. Among these, electrolyte regulation involves adding additives to the electrolyte to mitigate adverse reactions such as zinc anode dissolution, zinc dendrite formation, and corrosion. Previous studies have explored hybrid organic/aqueous systems, water-in-salt (WIS) electrolytes, semi-solid/hydrogel-type electrolytes, and solid-state electrolytes [9,10,11,12,13,14]. For instance, Xiang et al. organically combined polyacrylamide (PAM) and sodium alginate (SA) to fabricate a mechanically reinforced, low-water-content PAM/SA hydrogel enriched with anion-rich chains. This system demonstrated an ultrahigh ionic conductivity of 3.2 × 10^−2^ S cm^−1^, achieving ≥92% suppression of side reactions and regulated (002) crystal plane-oriented zinc deposition [15]. However, the WIS electrolyte modification strategy faces fundamental limitations in technological development due to its low conductivity, high viscosity, and salt precipitation issues. Additionally, inevitable salt crystallization at low temperatures narrows its operational temperature range [16]. In contrast, Dou et al. proposed a novel acetonitrile-assisted water-in-salt (AWIS) composite electrolyte strategy, creating a hybrid organic/aqueous electrolyte with LiTFSI salt. The AWIS electrolyte significantly improved conductivity, lowered freezing temperatures, reduced viscosity, and enhanced specific capacitance, rate capability, and low-temperature performance without compromising voltage stability or cycling stability [17]. Meanwhile, Gao et al. [18] introduced zinc formate as an organic small-molecule additive into the electrolyte. The free formate ions restructured the Zn^2+^ solvation sheath to accelerate Zn^2+^ transport, reducing zinc deposition overpotential to 28 mV. This enabled ultra-stable cycling for 2400 h (0.008% capacity decay per cycle) at 5 mA cm^−2^ in symmetric cells, along with extended stable cycling performance under high current density.

Recently, significant advances have been made in electrolyte additive design and interfacial engineering for AZIBs. For example, Pradeep Kumar Panda et al. systematically introduced novel organic/inorganic hybrid electrolyte systems and modification strategies, providing a comprehensive overview of functional additive selection for aqueous zinc-ion batteries and their effects on suppressing side reactions and dendrite formation, as well as enhancing cycling stability and reversibility [19]. These advances collectively highlight that the development of high-performance AZIBs is intrinsically linked to the rational engineering of electrolyte additives, as demonstrated by recent studies employing diverse additive molecules. For instance, carboxyl and hydroxyl functionalized organic molecules have been shown to strongly adsorb on zinc surfaces, forming a dehydrated inner Helmholtz layer that facilitates dendrite-free Zn deposition and suppresses side reactions. Incorporation of lactobionic acid (LA) as an additive led to a Zn plating/stripping average Coulombic efficiency of up to 99.89% (Zn//Cu, 1 mA cm^−2^, 1200 cycles) and enabled Zn//Zn symmetric cells to operate stably at 5 mA cm^−2^ for 1080 h [20]. Protein-based amyloid fibril (AF) additives create a hierarchical ion-transporting network and stably regulate local pH, markedly enhancing reversibility by enabling Zn||Zn symmetric batteries to cycle for 2500 h at 1 mA cm^−2^, as well as yielding remarkable capacity retention after 30,000 cycles in Zn-I2 systems [21]. Small organic molecules such as zinc formate can construct a stable Helmholtz electric double layer (HEDL), modulate Zn^2+^ solvation, and greatly increase hydrogen evolution overpotential. This results in symmetric Zn cells cycling for over 2400 h at 5 mA cm^−2^ and a Coulombic efficiency of 99.8% for Zn||VO_2_ batteries after 800 cycles [18]. In another typical example, a bifunctional zinc gluconate electrolyte delivers a robust Zn/electrolyte interface that inhibits dendrite growth, enabling symmetric battery cycling stability above 400 h and maintaining Coulombic efficiency over 95% for 140 cycles at 0.3 M additive concentration [22]. Collectively, these leading strategies underline the critical importance of additive structure and concentration in determining AZIB cycling performance and reversibility, and provide direct benchmarks to assess the properties of our proposed KG_0.15_ + DMSO_10_ system. These findings are summarized in Table 1.

Owing to their cost-effectiveness and eco-friendly characteristics, mixed aqueous/organic electrolyte systems have attracted considerable attention. Building upon this background, this study proposes a “synergistic solvation shell–interfacial adsorption regulation” strategy, in which 0.15 mol L^−1^ potassium gluconate (KG) and 10 vol% dimethyl sulfoxide (DMSO) are introduced as composite additives to simultaneously optimize the Zn^2+^ solvation structure and the interfacial stability of the zinc anode. DMSO efficiently integrates into the Zn^2+^ solvation shell, thereby weakening the interactions between Zn^2+^ and water molecules, reducing the activity of free water, and suppressing hydrogen evolution and corrosion side reactions. Meanwhile, gluconate anions preferentially adsorb onto the zinc anode surface, inducing the formation of a robust solid electrolyte interphase (SEI) that is enriched in inorganic components such as Zn(OH)_2_ and ZnCO_3_, which effectively inhibits zinc dendrite growth and mitigates parasitic reactions. Nuclear magnetic resonance (NMR) and Raman spectroscopy results demonstrate that the addition of KG_0.15_ + DMSO_10_ reduces the coordination number of water molecules around Zn^2+^, resulting in a reconfigured solvation structure; Fourier transform infrared spectroscopy(FTIR) further confirms the strong interactions between DMSO and water molecules. X-ray photoelectron spectroscopy (XPS) analysis reveals prominent O-C=O signals in the C 1s spectra and a significant attenuation of the O-H signal in the O 1 s spectra of the cycled zinc anode, indicating that the additives actively participate in the formation of the interphase layer. Benefiting from this synergistic regulation strategy, Zn||Zn symmetric cells demonstrate remarkably stable cycling for over 1800 h at 1 mA cm^−2^ and 1 mAh cm^−2^, far superior to the cells employing pure ZnSO_4_ electrolyte, and Zn||Cu cells achieve an average coulombic efficiency of 96.39% at 1 mA cm^−2^. This strategy provides a cost-effective and innovative approach for realizing high-performance aqueous zinc metal batteries.

## 2. Results and Discussion

Zn||Zn symmetric cells and Zn||Cu half-cells were assembled using electrolytes with varying additive concentrations and subjected to cycling stability and Coulombic efficiency (CE) tests under 1 mA cm^−2^ current density and 1 mAh cm^−2^ areal capacity. The results are presented in Figures S1 and S2. The stability of the electrolytes was evaluated through galvanostatic charge–discharge (GCD) testing of Zn||Zn symmetric cells. As shown in Figure S1, the polarization voltage and cycling stability of the Zn||Zn symmetric cells exhibited no significant dependence on additive concentrations, with minimal performance variations across different formulations. To further assess the reversibility of Zn^2+^ deposition/stripping during cycling, the CE of Zn||Cu half-cells was analyzed. Figure S2 compares the CE values of half-cells with different additive ratios. The half-cell employing the KG_0.15_ + DMSO_10_ electrolyte (0.15 mol L^−1^ potassium gluconate and 10 vol% dimethyl sulfoxide) demonstrated exceptional stability, maintaining a high and consistent CE (>96%) over 250 cycles. This performance markedly surpassed that of electrolytes with other additive concentrations. These results conclusively identify the KG_0.15_ + DMSO_10_ formulation as the optimal additive ratio for balancing reversibility, cycling stability, and suppression of side reactions.

Specifically, at lower KG concentrations (0.05 M and 0.1 M), the Coulombic efficiency (CE) exhibited more pronounced fluctuations and gradually declined during long-term cycling. When the KG concentration was increased to 0.2 M, CE stability was also reduced relative to the optimal condition. For DMSO content, both 5 vol% and 15 vol% produced lower CE values compared to 10 vol%, indicating that 10 vol% DMSO provides the best overall cycling performance. Based on these systematic investigations, the KG_0.15_ + DMSO_10_ electrolyte formulation was identified as optimal, as it delivers the highest and most stable CE over prolonged cycling, while simultaneously balancing the requirements for interfacial stability, reversibility, and suppression of side reactions.

**Figure 1:**
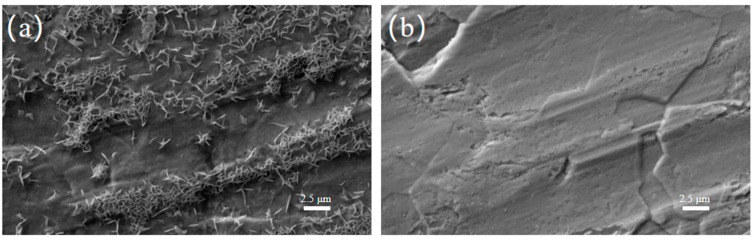
Surface morphology of zinc foils after 8 days of immersion in different electrolyte systems. (**a**) 2 M ZnSO_4_ electrolyte. (**b**) KG_0.15_ + DMSO_10_-modified electrolyte.

To visually explore the impact of the KG_0.15_ + DMSO_10_ electrolyte on the morphology and products of the zinc-ion battery anode, SEM was used to observe zinc foil soaked for 8 days in electrolytes with and without additives. Figure 1a shows that zinc foil soaked in pure ZnSO_4_ electrolyte grew many uneven zinc dendrites. In contrast, zinc foil soaked in the KG_0.15_ + DMSO_10_-added electrolyte had a smooth surface without noticeable dendrite growth or corrosion (Figure 1b) [23,24]. This indicates that Zn exhibits enhanced stability in the KG_0.15_ + DMSO_10_ electrolyte. This phenomenon may be attributed to the abundant gluconate ions present in KG_0.15_ + DMSO_10_, which form a robust adsorption layer on the Zn surface, effectively shielding it from corrosion by dissolved O_2_ [22].

**Figure 2:**
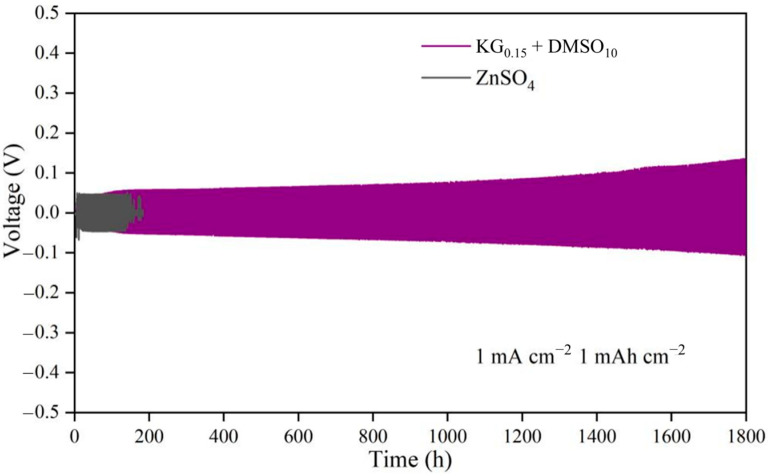
Cycling performance of Zn||Zn symmetric cells in electrolytes with and without additives at 1 mA cm^−2^ and 1 mAh cm^−2^.

Under testing conditions of 1 mA cm^−2^ and 1 mAh cm^−2^ (Figure 2), the Zn||Zn symmetric cell with the ZnSO_4_ electrolyte experienced short-circuiting after only 180 h of deposition/stripping cycles. This failure stems from the uneven Zn^2+^ deposition on the electrode surface, leading to zinc dendrite growth that pierced the separator [25]. In contrast, the Zn||Zn symmetric cell employing the KG_0.15_ + DMSO_10_ additive-modified electrolyte demonstrated exceptional stability, sustaining stable cycling for 1800 h—approximately 10 times longer than the baseline ZnSO_4_ system. This stark contrast highlights the critical role of additive modification in enhancing battery stability. To further evaluate the practical applicability of the additive-modified electrolyte, Zn||Zn symmetric cells were tested under high current densities of 3 mA cm^−2^/3 mAh cm^−2^ and 5 mA cm^−2^/5 mAh cm^−2^ (Figure S3). At 3 mA cm^−2^/3 mAh cm^−2^ (Figure S3a), the ZnSO_4_-based cell failed after merely 80 h, while the KG_0.15_ + DMSO_10_ electrolyte extended the cycling lifespan to 290 h. Similarly, at the extreme 5 mA cm^−2^/5 mAh cm^−2^ condition (Figure S3b), the additive-modified electrolyte achieved a significantly higher cycle life compared to the 91 h lifespan of the unmodified ZnSO_4_ system. These results conclusively demonstrate the additive-modified electrolyte’s superior capability to suppress dendrite growth and maintain structural integrity under demanding operational conditions.

**Figure 3:**
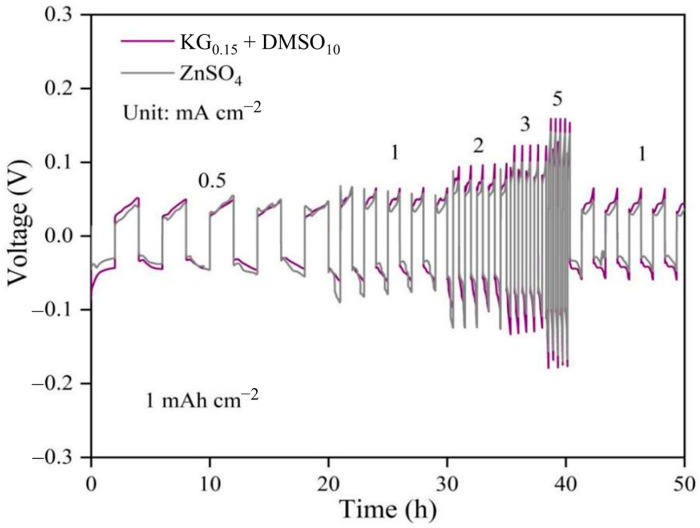
Rate performance and overpotential of Zn||Zn symmetric cells in different electrolyte systems.

To further validate the tolerance and voltage polarization of the additive-modified electrolyte under varying current densities, the rate performance of Zn||Zn symmetric cells was evaluated. As shown in Figure 3, even at a high current density of 5 mA cm^−2^, the additive-containing electrolyte exhibited a polarization voltage below 160 mV, marginally higher than the 142 mV observed in the baseline ZnSO_4_ system. This slight increase in polarization can be attributed to the modified solvation structure of Zn^2+^ ions and the elevated viscosity of the electrolyte induced by the additives, which influence the deposition/dissolution kinetics at the electrode interface. Notably, this moderate increase in overpotential is thermodynamically favorable for uniform zinc nucleation. Recent studies have shown that a moderately elevated surface overpotential reduces the critical nucleus size and lowers the nucleation barrier [26], promoting dense and homogeneous Zn deposition. Thus, the increased overpotential in our KG_0.15_ + DMSO_10_ electrolyte may contribute to finer nucleation and growth dynamics, potentially suppressing dendritic growth [27,28,29,30,31]. Additionally, the incorporation of DMSO and KG modulates the electrode/electrolyte interfacial properties, facilitating the formation of a stable interfacial layer on the zinc anode surface [32]. While this protective layer slightly increases polarization, the magnitude remains within tolerable limits. The trade-off between enhanced interfacial stability and moderate polarization growth underscores the effectiveness of the additive strategy in balancing performance and durability under high-rate conditions.

Notably, for Zn||Zn symmetric cells using the KG_0.15_ + DMSO_10_ electrolyte, the overpotential does not increase during the initial cycles. Instead, it rapidly reaches a stable value from the outset and remains constant upon further cycling, even at current densities of 1, 3, and 5 mA cm^−2^ (Figure 2 and Figure S3). This immediate stabilization reflects a dynamic equilibrium established at the interface, enabling extended cycling with minimal energy loss due to polarization. Importantly, the cycle life and durability of the symmetric cell are markedly improved, suggesting that the moderate increase in overpotential is offset by substantial gains in long-term stability and safety. Thus, although the energy efficiency is slightly reduced, the additive strategy provides an overall benefit for practical high-rate applications where cell lifespan and reliability are critical.

**Figure 4:**
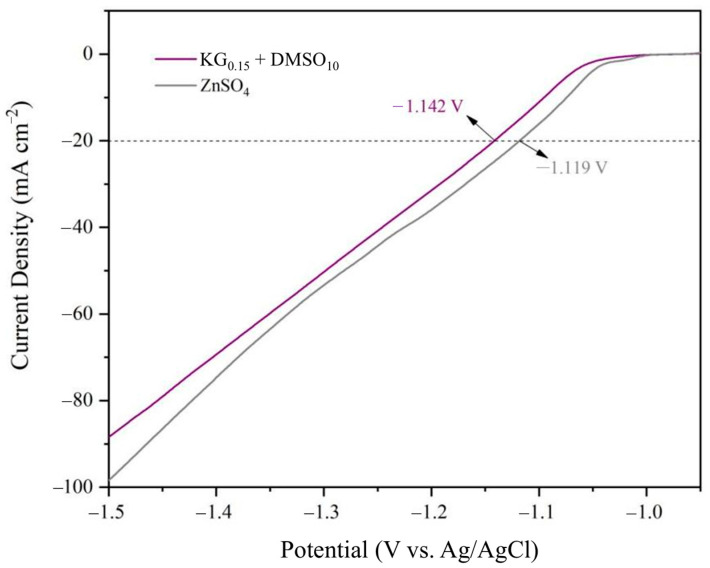
Linear sweep voltammetry(LSV) curves of different electrolytes in a three-electrode system.

Linear sweep voltammetry (LSV) was performed using a three-electrode system (zinc foil as the working electrode, platinum sheet as the counter electrode, and Ag/AgCl as the reference electrode) to investigate the hydrogen evolution overpotential of the zinc anode in different electrolytes. As shown in Figure 4, LSV analysis of the hydrogen evolution reaction (HER) revealed that at a current density of 20 mA cm^−2^, the additive-modified electrolyte exhibited a negative potential shift, expanding the stable electrochemical window from −1.119 V to −1.142 V. This demonstrates that the KG_0.15_ + DMSO_10_ additive-containing electrolyte effectively suppresses the HER during zinc deposition/stripping processes [33,34,35]. The widened electrochemical stability window underscores the additive’s role in mitigating parasitic hydrogen evolution, thereby enhancing the operational safety and longevity of aqueous zinc-ion batteries.

**Figure 5:**
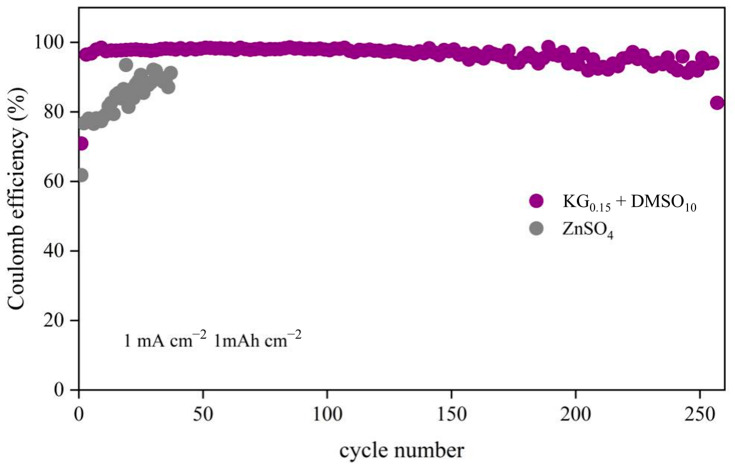
CE performance of Zn||Cu asymmetric cells in different electrolyte systems at 1 mA cm^−2^ and 1 mAh cm^−2^.

To further investigate the impact of the KG_0.15_ + DMSO_10_ additives on zinc anode stability, Zn||Cu half-cells were tested (Figure 5). The half-cell with the KG_0.15_ + DMSO_10_ electrolyte demonstrated stable Zn^2+^ deposition/stripping over 255 cycles, achieving an average Coulombic efficiency (CE) of 96.39%. In contrast, the ZnSO_4_-based half-cell exhibited significant voltage fluctuations from the outset and failed within 50 cycles, with an average CE of only 83.8%. The rapid failure of the unmodified system is attributed to uneven Zn^2+^ deposition, which promotes dendritic growth that pierces the separator, causing short circuits and continuous consumption of active zinc, thereby degrading capacity and CE [36,37]. The improved CE with additives confirms their role in regulating the electrode/electrolyte interface, enabling uniform and reversible Zn^2+^ deposition while suppressing dendrite formation and side reactions.

To validate the additive’s ability to homogenize zinc deposition and inhibit parasitic reactions, Zn||Zn symmetric cells with and without additives were subjected to 20 galvanostatic charge–discharge cycles, followed by XRD analysis of the zinc anodes (Figure S4). Post-cycling XRD patterns revealed a distinct peak at 2θ = 11.17° for the ZnSO_4_-based anode, corresponding to the formation of ZnSO_3_·2.5H_2_O byproducts. In contrast, no such byproduct peaks were observed in the additive-modified system, further confirming the suppression of undesirable side reactions at the zinc anode [38]. These results collectively underscore the critical role of KG_0.15_ + DMSO_10_ additives in enhancing interfacial stability and electrochemical reversibility. Furthermore, analysis of XRD intensity ratios for different crystal planes was performed, where the ratio of Zn(002) to Zn(101) is denoted as I_002_/I_101_, and that between the (002) and (100) planes as I_002_/I_100_. For bare zinc, the I_002_/I_100_ and I_002_/I_101_ values are 0.8 and 0.06, respectively, indicating a preferential growth along the (101) crystal plane. When ZnSO_4_ is used as the electrolyte, I_002_/I_100_ markedly increases to 7.79, and I_002_/I_101_ rises to 0.47, suggesting that Zn^2+^ tends to deposit along the (002) plane, resulting in highly oriented structures. However, the predominance of the (002) facet is associated with chemical instability, as it is readily corroded by water in aqueous electrolytes, leading to the formation of detrimental ZHS. This process promotes disordered Zn dendrite growth, poor CE, and limited lifespan of aqueous zinc metal batteries (AZMBs) [39]. Notably, upon the introduction of the KG_0.15_ + DMSO_10_ composite additive, I_002_/I_100_ and I_002_/I_101_ decrease to 2.88 and 0.29, respectively. This shift not only indicates effective suppression of excessive (002) plane-oriented growth, but also reflects the enhanced relative contribution of the (101) facet. Compared to (002), Zn growth along the (101) plane can maintain a stable vertical epitaxial mode and offers faster mass transfer kinetics, which is favorable for long-term and uniform regulation [40]. This advantageous behavior originates from the synergistic effect of KG and DMSO: Gluconate anions, with their multi-hydroxyl structure, preferentially adsorb onto the zinc surface, homogenizing the local electric field and inhibiting dendritic nucleation; DMSO, on the other hand, reconstructs the Zn^2+^ solvation shell and mitigates water activity, thereby reducing side reactions while optimizing ion transport pathways.

The interfacial Zn^2+^ transport in Zn||Zn cells was investigated using electrochemical impedance spectroscopy (EIS). Figure S5a,c show the Nyquist plots of Zn||Zn cells assembled with additive-containing and additive-free electrolytes after a 10 h rest period. The comparable interfacial resistances observed for both the KG_0.15_ + DMSO_10_-modified and pristine ZnSO_4_ electrolytes indicate that, in the initial state, no substantial interfacial layer has formed to affect electrode performance. In contrast, Figure S5d shows the Nyquist plot of the KG_0.15_ + DMSO_10_-based symmetric cell after 20 galvanostatic charge–discharge cycles, revealing two distinct semicircles. This emergence of an additional time constant in the medium-frequency range is attributed to the formation of an interfacial layer resulting from the interaction between the additives and the zinc anode during cycling. Compared with the ZnSO_4_ electrolyte, the Zn||Zn symmetric cell using the KG_0.15_ + DMSO_10_ electrolyte shows a higher charge transfer resistance (Figure S5d), mainly due to the fact that KG_0.15_ + DMSO_10_ suppresses the rapid Zn^2+^ transport during the desolvation process [41]. This kinetic modulation, while increasing interfacial resistance, is consistent with the formation of an evolved interface that contributes to improved electrochemical stability, as further demonstrated in the long-term cycling performance of symmetric.

To further evaluate the Zn^2+^ transport properties in the electrolyte, the Zn^2+^ transference number was measured for both ZnSO_4_ and KG_0.15_ + DMSO_10_ electrolyte systems. As shown in Figures S6 and S7, the Zn^2+^ transference number reaches 0.413 in the KG_0.15_ + DMSO_10_ system, significantly higher than that of pure ZnSO_4_ (0.219). This result supports the stable cycling performance of the Zn||Zn symmetric cell over 1800 h at 1 mA cm^−1^ and 1 mAh cm^−1^. The enhanced Zn^2+^ transference number provides strong electrochemical evidence for the superior cycling stability observed in this system.

**Figure 6:**
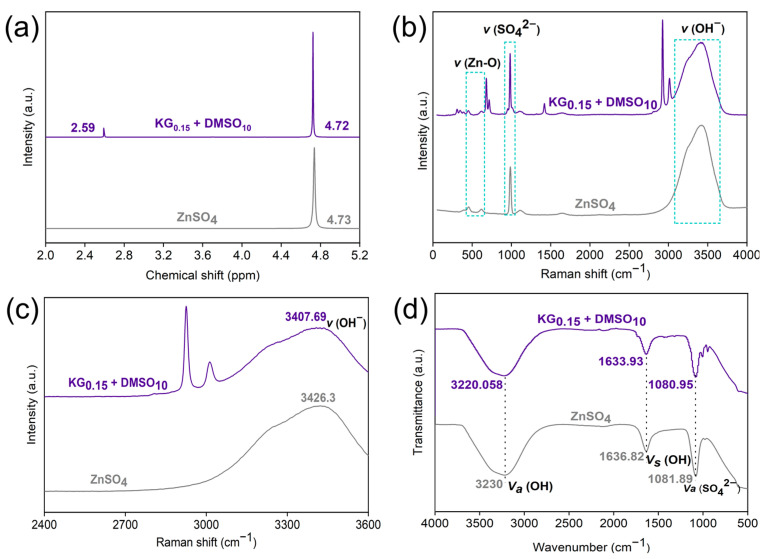
(**a**) ^1^H NMR spectra of the liquid phase for ZnSO_4_ and KG_0.15_ + DMSO_10_. (**b**) Raman spectra of the ZnSO_4_ and KG_0.15_ + DMSO_10_ electrolyte systems; (**c**) magnified Raman spectra in the 2400–3600 cm^−1^ region. (**d**) FTIR spectra.

To comprehensively elucidate the regulatory effect of the KG_0.15_ + DMSO_10_ additive on the Zn^2+^ solvation structure, NMR measurements were conducted. As shown in Figure 6a, upon the introduction of KG_0.15_ + DMSO_10_, the ^1^H NMR peak shifts from 4.73 to 4.72 ppm. This shift indicates that the addition of KG_0.15_ + DMSO_10_ promotes the release of several coordinated H_2_O molecules from the solvation shell, thus weakening the interaction between Zn^2+^ ions and H_2_O molecules [42]. A distinct new peak appears at 2.59 ppm, corresponding to the methyl (-CH_3_) group of DMSO [43], confirming that the polar S=O moiety of DMSO can participate in Zn^2+^ coordination and directly contribute to the reconstruction of the solvation shell. The changes in the solvation structure of Zn^2+^ in the presence of KG_0.15_ + DMSO_10_ were further verified by Raman and Fourier transform infrared (FTIR) spectroscopy. Both Raman and FTIR spectra clearly display characteristic functional groups of the gluconate ion (-C=O, -C-O, -C-C, -CH_3_), indicating the presence of interactions between gluconate ions and Zn metal. This indicates the existence of interactions between gluconate ions and Zn metal. Given that gluconate ions preferentially adsorb parallel to the electrode surface, and characteristic functional groups of the gluconate ion are absent in pure ZnSO_4_, these results confirm the parallel adsorption of gluconate ions on the Zn surface [22]. As shown in Figure 6b, for the KG_0.15_ + DMSO_10_ electrolyte, the peak observed near ~500 cm^−1^ is attributed to the V(Zn-O) vibration in the Zn-H_2_O solvation structure [44]. In the KG_0.15_ + DMSO_10_ system, this peak is split into two components, originating from Zn-H_2_O and Zn-gluconate complexes, respectively [22]. This demonstrates that gluconate ions can also participate in the solvation structure of Zn^2+^. As shown in Figure 6c, after the addition of KG_0.15_ + DMSO_10_, the O-H stretching vibration band shifts to lower wavenumbers, providing strong evidence for the disruption of the hydrogen-bond network [45]. As depicted in Figure 6d, a slight blue shift in the O-H band is observed, which is attributed to the formation of hydrogen bonds between DMSO and H_2_O [46]. In addition, the activity of H_2_O is suppressed in the KG_0.15_ + DMSO_10_ electrolyte. The SO_4_^2−^ stretching vibration also exhibits a slight blue shift, which can be ascribed to the weakened electrostatic coupling between Zn^2+^ and SO_4_^2−^, indicating that DMSO participates in theZn^2+^ solvation shell [40].

**Figure 7:**
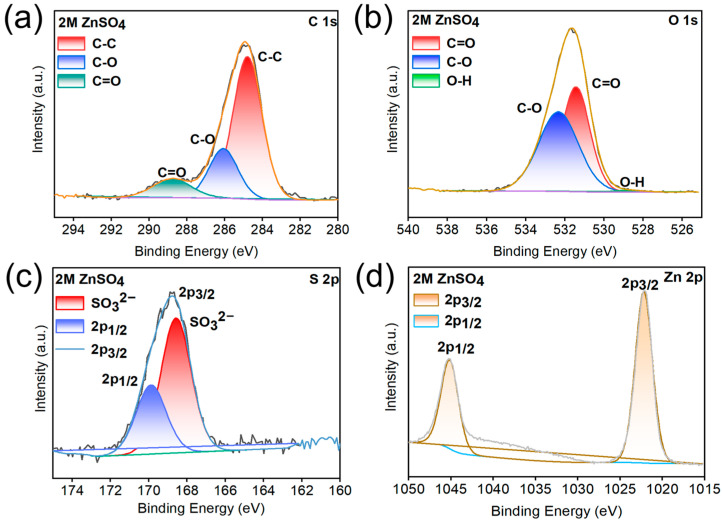
(**a**) C 1s, (**b**) O 1s, (**c**) S 2p and (**d**) Zn 2p XPS spectra of the Zn anode after 20 plating/stripping cycles in ZnSO_4_.

**Figure 8:**
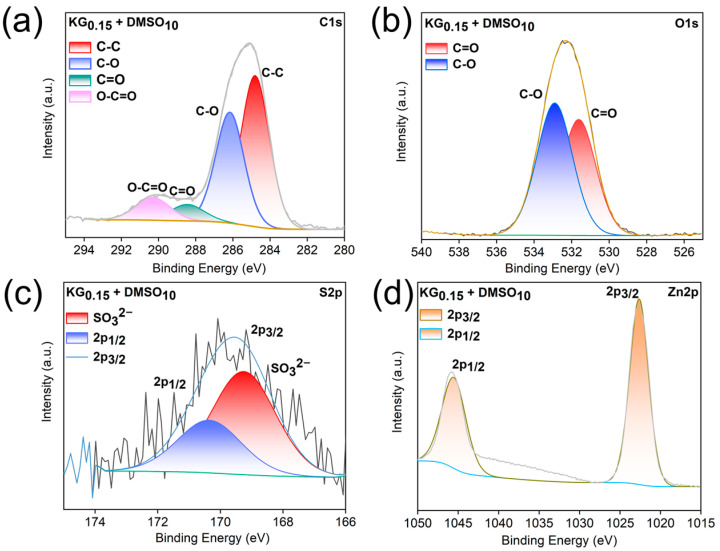
(**a**) C 1s, (**b**) O 1s, (**c**) S 2p and (**d**) Zn 2p XPS spectra of the Zn anode after 20 plating/stripping cycles in KG_0.15_ + DMSO_10_.

After 20 cycles at 1 mA cm^−2^ and 1 mAh cm^−2^, X-ray photoelectron spectroscopy (XPS) analyses of the Zn anodes in ZnSO_4_ and KG_0.15_ + DMSO_10_ electrolytes were conducted to investigate the adsorption behavior of KG_0.15_ + DMSO_10_ on the Zn surface. As shown in the C 1s spectra (Figure 7 and Figure 8), both samples exhibit relatively high carbon content. This is primarily attributed to the presence of inorganic carbonate species (CO_3_^2−^, mainly ZnCO_3_, with a binding energy at 289.1 eV), as well as C-O (286.2 eV) and C–C (284.8 eV) bonds from organic constituents. These components may originate from incompletely reduced Zn^2+^-anion complexes or residual salt ions [47,48,49,50]. After 20 cycles of plating/stripping with the addition of KG_0.15_ + DMSO_10_, the intensity of the C 1s peak increased significantly compared to that observed with the pure ZnSO_4_ electrolyte. This indicates the formation of a relatively thick solid electrolyte interphase (SEI) layer enriched in ZnCO_3_ [51]. Moreover, in the C 1s spectrum of the sample with KG_0.15_ + DMSO_10_ additive, a pronounced O–C=O characteristic peak is observed, which originates from the carboxyl groups (-COOH) in potassium gluconate [52]. Analysis of the O 1s spectra reveals that, after the addition of KG_0.15_ + DMSO_10_, the weak O-H peak present in the ZnSO_4_ electrolyte disappears. This phenomenon can be attributed to the formation of hydrogen bonds between water molecules and DMSO or the carboxyl groups (-COOH) in potassium gluconate, which reduces the number of free O-H bonds. Comprehensive analysis of the S 2p spectra confirms the presence of ZnSO_3_ in the sample, as evidenced by the peak located at 168.9 eV. ZnSO_3_ is formed upon decomposition of Zn^2+^-anion complexes. Previous studies have demonstrated that ZnSO_3_ can serve as an effective component of the solid electrolyte interphase (SEI), contributing to uniform Zn^2+^ deposition and suppressing water decomposition reactions. Moreover, the Zn 2p signal of the anode cycled in KG_0.15_ + DMSO_10_ exhibits a positive shift of 0.3 eV relative to that in ZnSO_4_, evidencing strong electronic interactions between the Zn anode and the adsorbed KG [53]. Collectively, the introduction of KG_0.15_ + DMSO_10_ establishes a uniform SEI enriched with ZnSO_3_ and Zn(OH)_2_, which shields the Zn electrode from severe side reactions and dendrite growth, thereby ensuring the long cycle life of AZIBs.

To evaluate the practical application of the KG_0.15_ + DMSO_10_ electrolyte in aqueous zinc-ion batteries, Zn||V_2_O_5_ full cells were assembled with V_2_O_5_ as the cathode. Figure S8 shows nearly identical CV curve shapes for full cells in both electrolytes, indicating similar electrochemical behavior in the two systems. However, the smaller CV curve area of the additive-modified full cell compared to the ZnSO_4_-based system suggests a lower initial capacity for the KG_0.15_ + DMSO_10_ electrolyte prior to cycling. This discrepancy can be attributed to the formation of an interfacial layer on the electrode surface induced by the additives, which consumes a portion of the active material during the initial cycles, thereby reducing the initial discharge capacity of the additive-containing full cell [54,55,56]. While this interfacial layer temporarily diminishes initial capacity, it plays a critical role in stabilizing the electrode/electrolyte interface, ultimately enhancing long-term cycling stability and suppressing degradation mechanisms.

**Figure 9:**
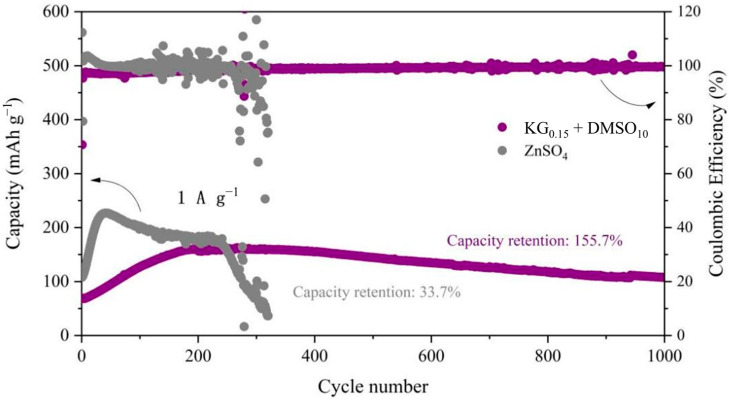
Long-cycle performance of Zn||V_2_O_5_ full cells in electrolytes with and without additives at 1A g^−1^.

**Figure 10:**
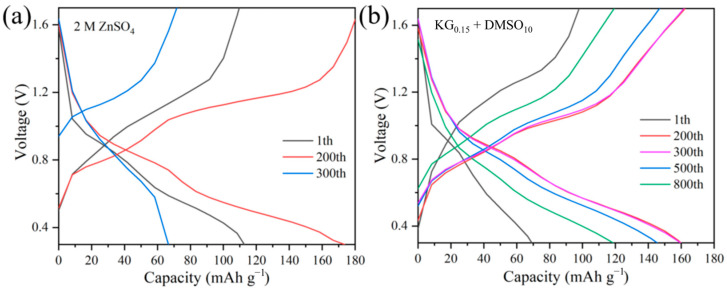
Voltage-capacity charge–discharge curves of Zn||V_2_O_5_ full cells in different electrolyte systems. (**a**) 2 M ZnSO_4_ electrolyte at cycles 1, 200, and 300. (**b**) KG_0.15_ + DMSO_10_ electrolyte at cycles 1, 200, 300, 500, and 800.

**Figure 11:**
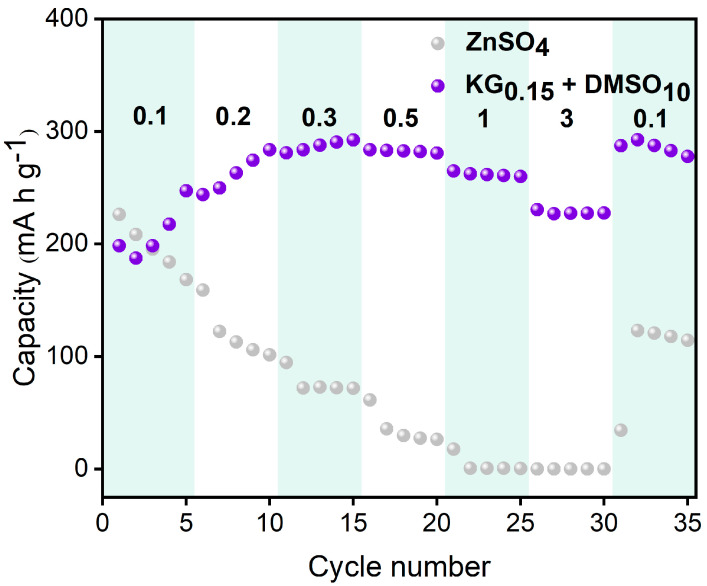
Rate performance of Zn||V_2_O_5_ full cells using different electrolytes.

To evaluate the influence of the KG_0.15_ + DMSO_10_ additive on the electrochemical performance of aqueous zinc-ion full batteries, Zn||V_2_O_5_ full cells were constructed (Figure 9). During the initial cycles, a gradual increase in cell capacity was observed, which is likely due to the activation of the V_2_O_5_ lattice. Post-activation, the number of active sites within V_2_O_5_ increases, thereby enhancing its ability to accommodate more Zn^2+^ ions [57]. The slightly lower initial discharge capacity in the KG_0.15_ + DMSO_10_ system, compared to the ZnSO_4_ baseline, can be attributed to the compatibility between the electrolyte and the electrode. Specifically, the addition of KG_0.15_ + DMSO_10_ alters the solvation structure of Zn^2+^, leading to a different interaction with the V_2_O_5_ positive electrode. This change in solvation structure may initially hinder the insertion/extraction kinetics of Zn^2+^ ions into/from the V_2_O_5_ lattice, resulting in a lower initial capacity [58]. However, after 250 cycles, the baseline ZnSO_4_ full cell suffers rapid capacity decay, while the additive-modified system shows a gradual capacity increase followed by stabilization (Figure 10b). Remarkably, the KG_0.15_ + DMSO_10_-based full cell retains a discharge capacity of 118.6 mAh g^−1^ after 800 cycles, whereas the ZnSO_4_ system plummets to 66.6 mAh g^−1^ by the 300th cycle (Figure 10a). After 1000 cycles, the additive-modified full cell achieves an exceptional capacity retention rate exceeding 100% (155.7%), vastly outperforming the baseline system’s 72.4% retention after 300 cycles. The rate performance of Zn||V_2_O_5_ full cells was further investigated at current densities ranging from 0.1 to 3 A g^−1^ (as shown in Figure 11). At the lower current density of 0.1 A g^−1^, both electrolyte systems exhibited similar charge/discharge capacities. However, as the current density increased, the cell with the KG_0.15_ + DMSO_10_ additive was able to maintain a higher capacity output. Notably, when the current density was returned to 0.1 A g^−1^, the cell using the KG_0.15_ + DMSO_10_ electrolyte recovered to a capacity of 292 mAh g^−1^, which is significantly higher than the 114 mAh g^−1^ observed for the cell with the pure ZnSO_4_ electrolyte.

## 3. Conclusions

In summary, the strategic incorporation of potassium gluconate (KG) and dimethyl sulfoxide (DMSO) as dual additives to modulate the Zn/electrolyte interface has been demonstrated as an effective approach for achieving high-performance aqueous zinc-ion batteries. Benefiting from its ability to restructure the solvation sheath, the KG_0.15_ + DMSO_10_-containing electrolyte significantly extends the cycling lifespan of Zn||Zn symmetric cells, achieving over 1800 h of stable operation under 1 mA cm^−2^ and 1 mAh cm^−2^, while enabling Zn||Cu half-cells to maintain an average Coulombic efficiency of 96.39% across 250 cycles. Furthermore, the KG_0.15_ + DMSO_10_ electrolyte exhibits exceptional compatibility with vanadium-based cathodes, substantially enhancing both the cycling stability and capacity retention of Zn||V_2_O_5_ full cells. These findings establish a novel electrolyte design paradigm that simultaneously addresses zinc dendrite suppression, parasitic reaction mitigation, and interfacial stability optimization. The work provides a viable and scalable solution for advancing the practical implementation of aqueous zinc-ion batteries in energy storage systems.

## Figures and Tables

**Table 1:** Performance comparison of the KG_0.15_ + DMSO_10_ electrolyte with representative electrolyte additives in aqueous zinc-ion batteries.

Additive	System	Current Density (mA cm^−2^)	Areal Capacity (mAh cm^−2^)	Cycle Life (h)	Coulombic Efficiency (%)	Ref.
Lactobionic acid (LA)	Zn||Zn/Zn||Cu	5/10	5/10	>1080/500	-/99.89	[20]
Protein-based AF	Zn||Zn	1/5	1/5	2500/500	-/99.7	[21]
Zinc formate	Zn||Zn/Zn||VO_2_	5/-	1/-	>2400/-	-/98.1	[18]
Zinc gluconate	Zn||Zn/Zn||Cu	0.1/-	0.1/-	>400/-	-/>95	[22]
KG_0.15_ + DMSO_10_ (this work)	Zn||Zn/Zn||Cu	1	1	>1800	>96	-

## Data Availability

The data presented in this study are available within the article and the Supplementary Materials in the form of figures and tables.

## Supplementary Materials

The following supporting information can be downloaded at: https://www.mdpi.com/article/10.3390/nano15191482/s1. Figure S1: Performance evaluation of Zn||Zn symmetric cells with different concentrations of potassium gluconate (KG) and dimethyl sulfoxide (DMSO) additives under 1 mA cm^−2^ and 1 mAh cm^−2^ test conditions; Figure S2: Coulombic efficiency (CE) performance plot of Zn||Cu half-cells with different additive concentrations of potassium gluconate (KG) and dimethyl sulfoxide (DMSO) under 1 mA cm^−2^ and 1 mAh cm^−2^ conditions; Figure S3: Constant-current cycling performance of Zn||Zn symmetric cells with/without additives under high current densities. (a) 3 mA cm^−2^, 3 mAh cm^−2^, and (b) 5 mA cm^−2^, 5 mAh cm^−2^; Figure S4: XRD patterns of the zinc anode in Zn||Zn symmetric cells after cycling in different electrolyte systems; Figure S5: Nyquist plots of Zn||Zn symmetric cells in different electrolyte systems before and after cycling. (a,b) Nyquist plots in 2 M ZnSO_4_ electrolyte, (c,d) Nyquist plots in electrolyte with KG_0.15_ + DMSO_10_ additives; Figure S6: I-t curve of the Zn||Zn symmetric cell in the ZnSO_4_ electrolyte; The insets show EIS spectra before and after polarization; Figure S7: I-t curve of the Zn||Zn symmetric cell in the KG_0.15_+DMSO_10_ electrolyte; The insets show EIS spectra before and after polarization. Figure S8. CV curves of the Zn||V_2_O_5_ full battery at a scan rate of 1 mV s^−1^.
