# Supplementary material for: Synergistic Regulation of Solvation Shell and Anode Interface by Bifunctional Additives for Stable Aqueous Zinc-Ion Batteries
Source: Nanomaterials (Basel). 2025 Sep 28;15(19):1482. doi: 10.3390/nano15191482 (PMC12525561; doi:10.3390/nano15191482)
Supplement: Supplementary file 1 [file nanomaterials-15-01482-s001.zip › nanomaterials-3884284-supplementary.pdf]

nanomaterials

MDPI

Supplementary Materials

Synergistic Regulation of Solvation Shell and Anode Interface by Bifunctional Additives for Stable Aqueous Zinc-Ion Batteries

Luo Zhang [1,2], Die Chen [1], Chenxia Zhao [1], Haibo Tian [2], Gaoda Li [3], Xiaohong He [3], Gengpei Xia [4,*], Yafan Luo [5,*] and Dingyu Yang [1,3,*]

[1] Sichuan Meteorological Optoelectronic Sensor Technology and Application Engineering Research Center, Chengdu University of Information Technology, Chengdu 610225, China
[2] Information Materials and Device Applications Key Laboratory of Sichuan Provincial Universities, Chengdu University of Information Technology, Chengdu 610225, China
[3] Optoelectronic Sensor Devices and Systems Key Laboratory of Sichuan Provincial Universities, College of Optoelectronic Engineering (Chengdu IC Valley Industrial College), Chengdu University of Information Technology, Chengdu 610225, China
[4] Chengdu Product Quality Inspection and Research Institute Co., Ltd., Chengdu 610100, China
[5] POWERCHINA Chengdu Engineering Corporation Limited, Chengdu 611130, China
* Correspondence: xiagp@cqi.org (G.X.); p2020337@chidi.com.cn (Y.L.); yangdingyu@cuit.edu.cn (D.Y.)

Academic Editor: Diego Cazorla-Amorós

Received: 4 September 2025
Revised: 23 September 2025
Accepted: 23 September 2025
Published: 26 September 2025

Citation: Zhang, L.; Chen, D.; Zhao, C.; Tian, H.; Li, G.; He, X.; Xia, G.; Luo, Y.; Yang, D. Synergistic Regulation of Solvation Shell and Anode Interface by Bifunctional Additives for Stable Aqueous Zinc-Ion Batteries. *Nanomaterials* **2025**, *15*, x. https://doi.org/10.3390/xxxxx

Copyright: © 2025 by the authors. Submitted for possible open access publication under the terms and conditions of the Creative Commons Attribution (CC BY) license (https://creativecommons.org/licenses/by/4.0/).

S1. Experimental section

S1.1. Preparation of Electrolyte

An appropriate amount of $ZnSO_4{\cdot}7H_2O$ powder was dissolved in deionized water and magnetically stirred at room temperature for 2 h to prepare a 2 mol L^{-1} $ZnSO_4$ electrolyte (denoted as $ZnSO_4$). Subsequently, potassium gluconate (KG) was added to the 2 M $ZnSO_4$ solution as the base solvent, followed by magnetic stirring for 2 h at room temperature, to prepare KG solutions with varying concentrations (referred to as KGx, where x = 0.01, 0.1, 0.15, and 0.2 mol L^{-1}). Finally, dimethyl sulfoxide (DMSOy, where y = 5, 10, and 15 vol%) was introduced into the KGx-containing electrolytes at different volume ratios. The mixtures were magnetically stirred for 2 h at room temperature to obtain the final hybrid electrolytes, designated as KG_x + $DMSO_y$.

S1.2. Electrochemical Measurement

Zn||Zn symmetric cells, Zn||Cu half-cells, and Zn||V_2O_5 full cells were assembled using CR2016 coin cells with 200 μL of electrolyte added to each cell. The cells employed glass fiber (GF/D) separators and utilized either $ZnSO_4$ or $KG_{0.15}$ + $DMSO_{10}$ as the electrolyte. Galvanostatic charge–discharge (GCD) testing was performed on the Zn||Zn, Zn||Cu, and Zn||V_2O_5 configurations under varying current densities using a Neware testing system.

Cyclic voltammetry (CV) and electrochemical impedance spectroscopy (EIS) measurements were conducted on a BioLogic SP-150 electrochemical workstation. CV tests were performed on Zn||V_2O_5 full cells within a voltage range of 0.3 V to 1.7 V at a scan rate of 1 mV s^{-1}. EIS tests were carried out on Zn||Zn symmetric cells over a frequency range of 0.01 Hz to 100 kHz.

Linear sweep voltammetry (LSV) was executed using a Corrtest Instrument equipped with a three-electrode system to investigate the impact of additives on the hydrogen evolution reaction (HER) at the zinc anode. A zinc foil served as the working elec-

trode, a platinum electrode as the counter electrode, and an Ag/AgCl electrode as the reference electrode. The LSV tests were conducted at a scan rate of 1 mV s^{-1} across a voltage range of −0.9 V to −1.5 V.

Zinc Ion Transference Number was determined by combining electrochemical impedance spectroscopy (EIS) and chronoamperometry (CA) on a Zn||Zn symmetric cell. EIS measurements were performed over a frequency range of 10 mHz to 100 kHz; the chronoamperometry measurement was implemented by imposing a bias voltage of 20 mV for 6000 s, followed by another AC impedance measurement. The Zn^{2+} transference number (t) was calculated using the following equation:

$$t_{Zn^{2+}} = \frac{I_S(\Delta V - I_0 R_0)}{I_0(\Delta V - I_S R_S)} \quad \text{(S1)}$$

S1.3. Material Characterization

The morphologies of zinc anodes after reaction in different electrolytes were observed using scanning electron microscopy (SEM; Hitachi SU8020, Hitachi High-Tech Corporation, Tokyo, Japan), and the crystal structures of the zinc anodes were analyzed by X-ray diffraction (XRD; Bruker D8 Advance, Bruker AXS GmbH, Karlsruhe, Germany). XRD measurements were performed with Cu Kα radiation at a scan rate of 5°/min over a scanning angle range of 5° to 90°. Chemical states of the electrode surface were analyzed by X-ray photoelectron spectroscopy (XPS; Thermo Scientific K-Alpha, Thermo Fisher Scientific, Waltham, MA, USA). Changes in the hydrogen-bond network and Zn^{2+} solvation structure were probed via Raman spectroscopy (Horiba LabRAM HR Evolution, HORIBA Scientific, Kyoto, Japan) and Fourier-transform infrared spectroscopy (FTIR; Thermo Fisher Nicolet iS20, Thermo Fisher Scientific, Waltham, MA, USA). Solvation structures of Zn^{2+} in electrolytes were investigated by liquid-state nuclear magnetic resonance (NMR; Bruker 400 MHz, Bruker AXS GmbH, Karlsruhe, Germany).

S2. Supporting Figures

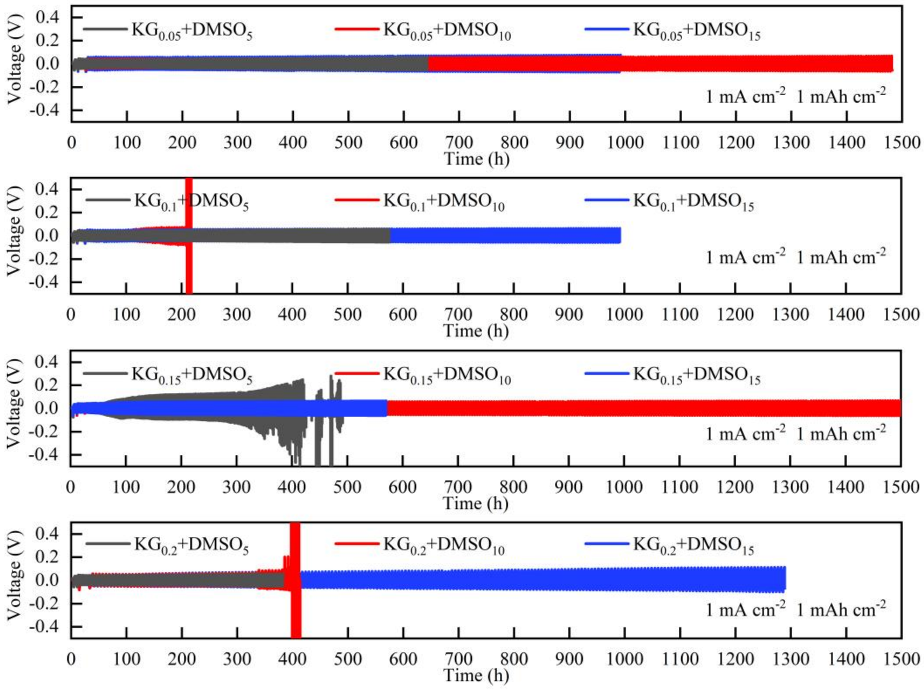

Figure S1. Performance evaluation of Zn||Zn symmetric cells with different concentrations of potassium gluconate (KG) and dimethyl sulfoxide (DMSO) additives under 1 mA cm⁻² and 1 mAh cm⁻² test conditions.

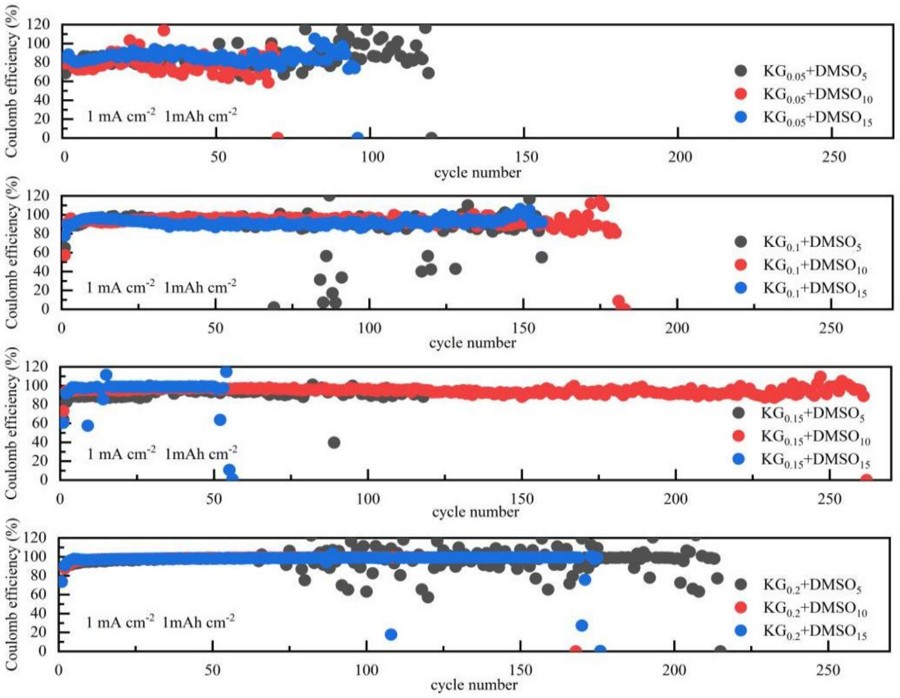

Figure S2. Coulombic efficiency (CE) performance plot of Zn||Cu half-cells with different additive concentrations of potassium gluconate (KG) and dimethyl sulfoxide (DMSO) under 1 mA cm⁻² and 1 mAh cm⁻² conditions.

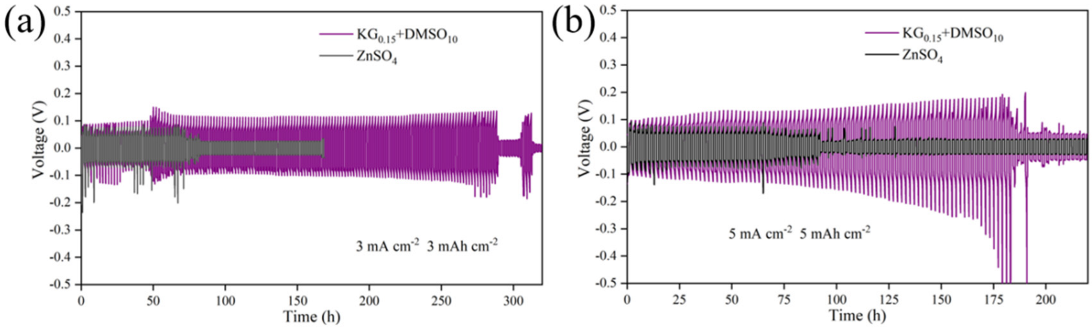

Figure S3. Constant-current cycling performance of Zn||Zn symmetric cells with/without additives under high current densities.(**a**) 3 mA cm⁻², 3 mAh cm⁻²,and (**b**) 5 mA cm⁻², 5 mAh cm⁻².

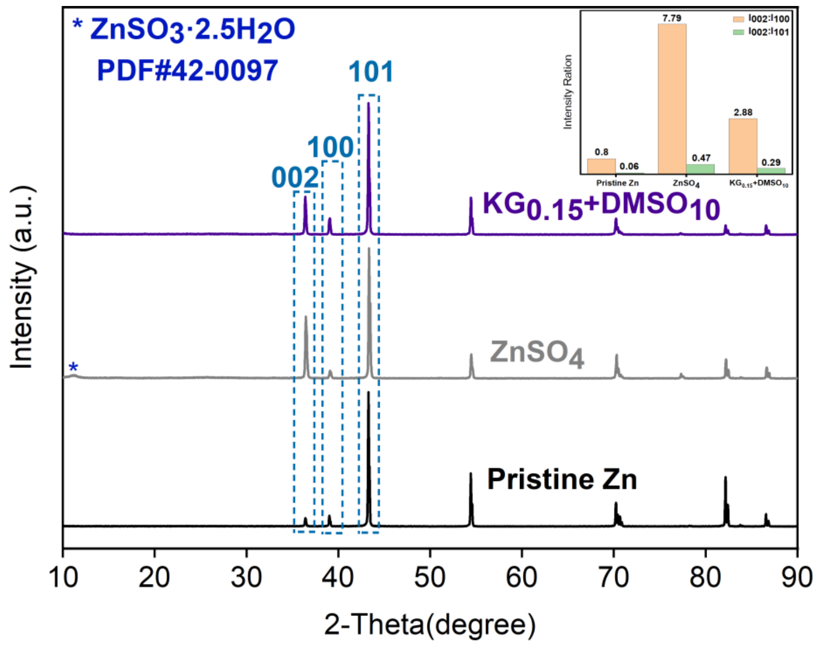

Figure S4. XRD patterns of the zinc anode in Zn||Zn symmetric cells after cycling in different electrolyte systems.

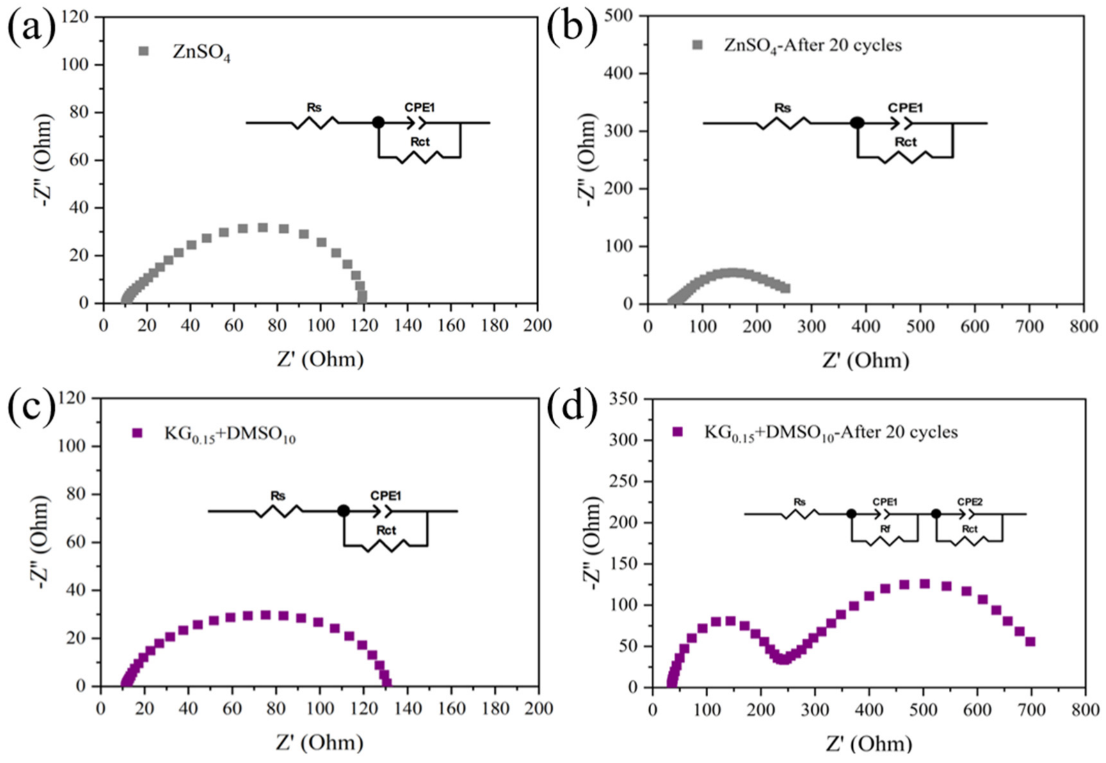

Figure S5. Nyquist plots of Zn||Zn symmetric cells in different electrolyte systems before and after cycling. (**a**,**b**) Nyquist plots in 2 M $ZnSO_4$ electrolyte, (**c**,**d**) Nyquist plots in electrolyte with $KG_{0.15}$ + $DMSO_{10}$ additives.

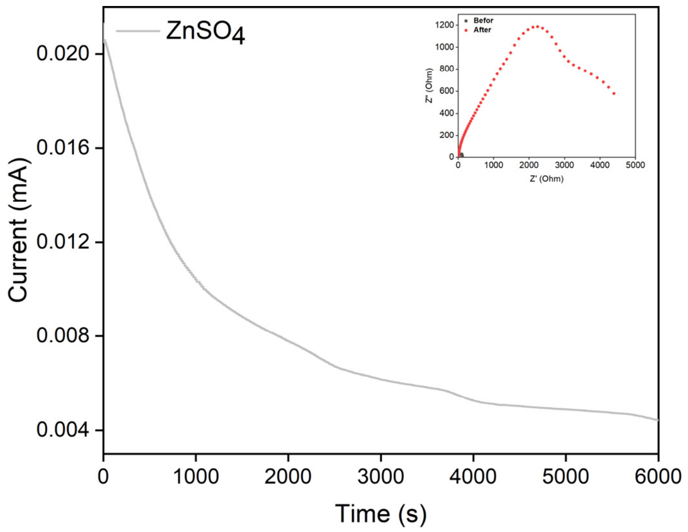

Figure S6. I-t curve of the Zn||Zn symmetric cell in the $ZnSO_4$ electrolyte; The insets show EIS spectra before and after polarization.

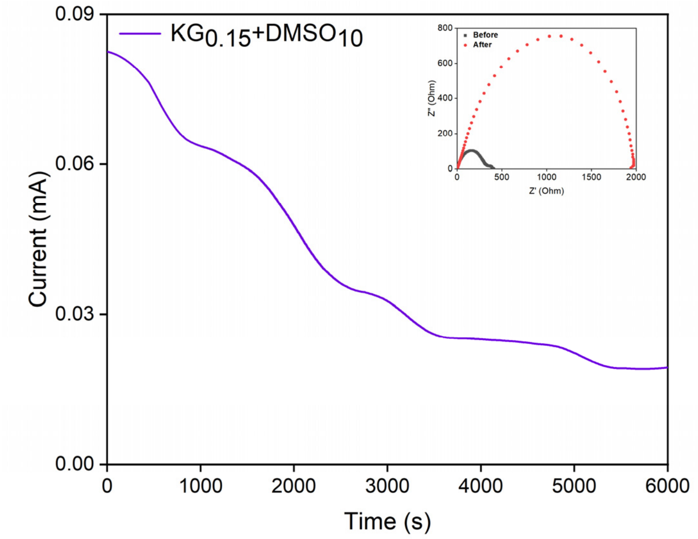

Figure S7. I-t curve of the Zn||Zn symmetric cell in the $KG_{0.15}$ + $DMSO_{10}$ electrolyte; The insets show EIS spectra before and after polarization.

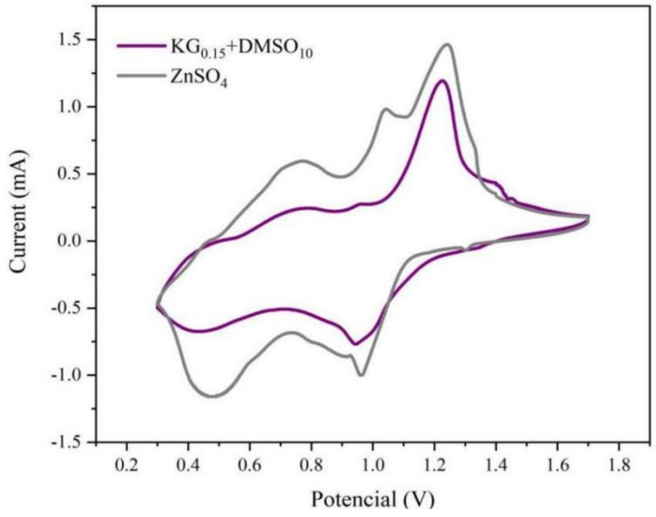

Figure S8. CV curves of the Zn||V_2O_5 full battery at a scan rate of 1 mV s^{-1}.
